# Late-Onset Cryopyrin-Associated Periodic Syndromes Caused by Somatic NLRP3 Mosaicism—UK Single Center Experience

**DOI:** 10.3389/fimmu.2017.01410

**Published:** 2017-10-31

**Authors:** Dorota M. Rowczenio, Sónia Melo Gomes, Juan I. Aróstegui, Anna Mensa-Vilaro, Ebun Omoyinmi, Hadija Trojer, Anna Baginska, Alberto Baroja-Mazo, Pablo Pelegrin, Sinisa Savic, Thirusha Lane, Rene Williams, Paul Brogan, Helen J. Lachmann, Philip N. Hawkins

**Affiliations:** ^1^National Amyloidosis Centre, University College London, London, United Kingdom; ^2^Great Ormond Street Institute of Child Health (ICH), University College London, London, United Kingdom; ^3^Department of Immunology, Hospital Clinic-IDIBAPS, Barcelona, Spain; ^4^Inflammation and Experimental Surgery Unit, Biomedical Research Institute of Murcia IMIB-Arrixaca, Clinical University Hospital Virgen de la Arrixaca, Murcia, Spain; ^5^National Institute for Health Research–Leeds Musculoskeletal Biomedical Research Unit, Leeds Institute of Rheumatic and Musculoskeletal Medicine, Leeds, United Kingdom

**Keywords:** cryopyrin-associated periodic syndrome, ASC aggregates, AA amyloidosis, NLRP3 somatic mutation, mutant allele, IL-1β

## Abstract

Cryopyrin-associated periodic syndrome (CAPS) is caused by *gain-of-function NLRP3* mutations. Recently, somatic *NLRP3* mosaicism has been reported in some CAPS patients who were previously classified as “mutation-negative.” We describe here the clinical and laboratory findings in eight British adult patients who presented with symptoms typical of CAPS other than an onset in mid-late adulthood. All patients underwent comprehensive clinical and laboratory investigations, including analysis of the *NLRP3* gene using Sanger and amplicon-based deep sequencing (ADS) along with measurements of extracellular apoptosis-associated speck-like protein with CARD domain (ASC) aggregates. The clinical phenotype in all subjects was consistent with mid-spectrum CAPS, except a median age at disease onset of 50 years. Sanger sequencing of *NLRP3* was non-diagnostic but ADS detected a somatic *NLRP3* mutation in each case. In one patient, DNA isolated from blood demonstrated an increase in the mutant allele from 5 to 45% over 12 years. ASC aggregates in patients’ serum measured during active disease were significantly higher than healthy controls. This series represents 8% of CAPS patients diagnosed in a single center, suggesting that acquired *NLRP3* mutations may not be an uncommon cause of the syndrome and should be sought in all patients with late-onset symptoms otherwise compatible with CAPS. Steadily worsening CAPS symptoms in one patient were associated with clonal expansion of the mutant allele predominantly affecting myeloid cells. Two patients developed AA amyloidosis, which previously has only been reported in CAPS in association with life-long germline *NLRP3* mutations.

## Introduction

Cryopyrin-associated periodic syndrome (CAPS) is a dominantly inherited autoinflammatory disease (AID) comprising three overlapping clinical entities of varying severity: familial cold autoinflammatory syndrome (FCAS) represents the mildest phenotype, Muckle–Wells syndrome (MWS) is of intermediate severity, and chronic infantile, neurological, cutaneous, and articular (CINCA) syndrome, also known as neonatal-onset multisystem inflammatory disease (NOMID), is most severe (1–5). The disease typically presents at birth or early infancy, characterized by recurrent episodes of fever, urticaria-like rash, arthralgia, myalgia, aseptic meningitis, and inflammatory eye manifestations. Up to 60% of patients with MWS suffer from progressive sensorineural hearing loss, usually beginning in adolescence, and up to 25% eventually develop AA amyloidosis as a complication of severe longstanding inflammation, which leads to nephrotic syndrome, renal failure, and death (2).

Cryopyrin-associated periodic syndrome is caused by mutations in the *NLRP3* gene leading to enhanced activation of the NLRP3-inflammasome and overproduction of IL-1β (2–5). Typically, activation of the NLRP3-inflammasome occurs *via* two-signal model and numerous stimuli are known to be the trigger (6). The gain-of-function mutations in the *NLRP3* gene decrease the threshold to activate the NLRP3-inflammasome by means of the reduction to only one stimulus (7).

Autosomal-dominant inheritance is readily apparent in about 75% of patients with MWS and FCAS whereas *de novo NLRP3* mutations account for CINCA/NOMID in more than half of affected children. The apparently paradoxical absence of an *NLRP3* mutation using traditional Sanger technology in other children with CINCA/NOMID has lately been elucidated in some cases through the application of highly sensitive genetic methods, including PCR-cloning and amplicon-based deep sequencing (ADS). Several studies have identified somatic mosaicism in the *NLRP3* gene in children with CINCA/NOMID (8–11) and MWS (12), consistent with a mutation occurring during embryogenesis. However, *NLRP3* mosaicism has lately also been reported in a 52-year-old woman and a 62-year-old man with recent onset of CAPS symptoms (13, 14), suggesting the mutational event occurred late in life. *NLRP3* mosaicism can thus be associated with a spectrum of CAPS phenotypes and age at disease onset. An acquired CAPS-like disorder known as Schnitzler syndrome can very rarely develop in patients with low grade lymphoplasmacytic lymphoma, characterized by the presence of an IgM paraprotein and, intriguingly, myeloid-restricted somatic *NLRP3* mosaicism has been reported in two patients who had been diagnosed to have Schnitzler syndrome (15).

We report here eight patients with somatic *NLRP3* mosaicism, who presented in mid to late adulthood with symptoms consistent with CAPS. None of these patients had a family history of similar illness and all had been completely healthy previously. Their clinical features and response to treatment are described in detail. To investigate the etiology and dynamics of the mutational event in our cohort, we studied samples from multiple tissues and were able to perform a time-course study in one patient.

## Patients and Methods

Between 2005 and 2016, a clinical diagnosis of CAPS was supported by genetic findings in 100 adult patients referred to the UK National Amyloidosis Centre (NAC). In eight further subjects, the clinical diagnosis was not supported by Sanger sequencing; these patients subsequently underwent ADS analysis of the *NLRP3* gene to investigate the possibility of mosaicism. All subjects underwent comprehensive clinical assessment including search for a monoclonal immunoglobulin, including immunofixation of serum and urine to exclude Schnitzler syndrome, serial measurements of the acute phase reactants [serum amyloid A protein (SAA) and C-reactive protein (CRP)], and screening for AA amyloidosis on a renal biopsy and by clinical examination, renal function tests, and ^123^SAP scintigraphy.

Patients were asked to complete the QualityMetric SF36v2^®^ Health Survey designed to measure functional health and well-being from the patient’s perspective before starting treatment and at various time-points on treatment.

Informed consent was provided by all subjects, and the ethical approval for the study was obtained from Royal Free Hospital and University College Medical School Research Ethics Committee for this retrospective study (REC reference number 06/Q0501/42) and was in accordance with the Declaration of Helsinki.

### Molecular Investigations in the Eight “Mutation-Negative” CAPS Patients

DNA was isolated from whole blood in all patients. In six, we were able to obtain additional samples, including saliva, buccal epithelial cells, urine, fresh blood for isolation of circulating lymphocytes, and myeloid cells. Isolation of neutrophils, monocytes, and T and B lymphocytes was performed with magnetic nanoparticles (Stemcell Technologies, Inc., Manchester, UK) following the manufacturer’s protocol. DNA was extracted from isolated cells using Qiagen Investigator DNA extraction Kit (Qiagen Ltd., Manchester, UK).

*NLRP3/CIAS1* (exons 3, 4, and 6) [NCBI RefSeqGene NC_000001.10 (LRG_197)] and *NLRP12* (exon 3) [NCBI RefSeqGene NC_000019.9] genes were amplified by PCR and analyzed by Sanger sequencing using the protocol described previously (16). The chromatograms were analyzed on the ABI 3130xl Genetic Analyser using Sequencing Analysis Software version 5.4.

Amplicon-based deep sequencing was performed to amplify all exons of the *NLRP3* gene using specific pair primers as previously described (17). All PCR amplicons were deep sequenced (mean coverage 3,500×) on different platforms (GS Junior 454, PGM IonTorrent and Illumina MiSeq). The sequences were analyzed using the Amplicon Variant Analyzer software (Roche, Switzerland).

### Detection of ASC Protein Aggregates and IL-1β in Serum

Extracellular ASC protein aggregates were measured as described elsewhere (Baroja-Mazo, 2014 #6909). Briefly, 400 µl of plasma collected from 6 patients was incubated with 1 µg of rabbit polyclonal anti-ASC (Adipogen) at room temperature for 2 h, and another 400 µl of the same plasma samples were incubated in parallel with 1 µg of rabbit normal IgG, as non-specific staining control. Serum was centrifuged (2,500 × *g* × 8 min), washed and the pellets were incubated for 1 h with 0.5 µg of anti-rabbit IgG Alexa Fluor 647 (Life Technologies). All samples were analyzed using a FACSCanto flow cytometer (BD) and FACSDiva software (BD) by gating for small particles based on forward scatter versus side scatter. A number of ASC protein aggregates per milliliter of serum were calculated after subtraction of non-specific staining samples. IL-1β was also quantified in the serum samples using a specific ELISA for human IL-1β (R&D), following the manufacturer instructions.

### Histology and Immunohistochemistry

Six-micrometer-thick sections of kidney biopsies from patients 3 and 4 who presented with nephrotic syndrome were stained for amyloid with Congo red and viewed under crossed polarized light (18). Confirmation of AA-type amyloid deposits was sought immunohistochemically using monoclonal antibodies specific to SAA (Euro-Diagnostica).

### Radiolabeled Serum Amyloid P Component (SAP) Scintigraphy

Those two patients diagnosed with AA amyloidosis underwent whole body anterior and posterior scintigraphic imaging 24 h after administration of ^123^I-labeled SAP using a GE Infinia Hawkeye gamma camera, as previously described. The SAP results were interpreted by a panel of physicians with experience of over 30,000 SAP scans (19).

## Results

### Clinical Characteristics in the Eight Subjects with Somatic *NLRP3* Mutations

The clinical features in this cohort are summarized in Table 1. The median age at disease onset was 50 years (range 31–71 years); median age at clinical diagnosis with CAPS was 65 years (range 51–79 years) and the median duration of symptoms prior to referral was 15 years (range 8–20 years). All patients suffered with urticaria-like rash, which was most prominent on the trunk and legs, accompanied by fatigue and fevers and appeared to be much worse in the evenings and exacerbated by exposure to cold. All subjects had been diagnosed with progressive bilateral sensorineural hearing loss; four had conjunctivitis, three complained of frequent headaches, three had papilledema and one had optic neuritis. Baseline measurement revealed elevated inflammatory markers in all. Two patients developed AA amyloidosis complicated by nephrotic syndrome prior to their referral to our center. None of these subjects had a family member suffering with similar symptoms. A potential diagnosis of Schnitzler syndrome was pursued in all cases but was excluded by absence of a circulating paraprotein.

**Table 1:** Clinical characteristics in the eight patients with late onset of cryopyrin-associated periodic syndrome caused by somatic mosaicism in the *NLRP3* gene.

Patient	Clinical symptoms	Duration of symptoms (years)/age at diagnosis (years)	Median Pre-treatment SAA/CRP (mg/l)	DNA substitution/protein variant *(NCBI Ref Seq: NM_001243133.1)*	MAF mean (%)	Coverage of mutation mean (X)
1	UR, HF, BSD, conjunctivitis, headaches, papilledema	20/65–70	415/82	c.1688A > G/p.Y563C	5.1	1,994
2	UR, HF, BSD, arthralgia, headaches, nausea, diarrhea, and marked lymphadenopathy	10/60–65	446/53	c.1688A > G/p.Y563C	3.2	11,969
3	UR, HF, BSD, iritis, optic neuritis, papilledema, nephrotic syndrome, weight loss, AA amyloidosis	10/60–65	473/162	c.1688A > G/p.Y563C	11.1	1,085
4	UR, HF, BSD, abdominal pain, fatigue, bilateral clubbing, nephrotic syndrome, weight loss, AA amyloidosis	20/65–70	79/42	c.1054G > A/p.A352T	14.6	6,738
5	UR, HF, BSD, lymphadenopathy, conjunctivitis, died of pancreatic cancer	8/75–80	397/108	c.1706G > T/p.G569V	21.1	2,535
6	UR, HF, BSD, conjunctivitis, fatigue, arthralgia, headaches, bilateral clubbing; papilledema chronic aseptic meningitis	10/50–55	121/54	c.1699G > A/p.E567K	5.4	1,293
7	UR, HF, BSD, arthralgia and myalgia, conjunctivitis, and severe headaches.	20/50–55	276/291	c.1700 G > C/p.E567Q	15	4,612
8	UR, HF, BSD, headaches, myalgia	15/65–70	Not done/146	c.1691G > A/p.G564D	5.0	2,592

*UR, urticaria-like rash induced by cold; HF, high-grade fever; BSD, bilateral sensorineural deafness; MAF, mutant allele frequency; SAA, serum amyloid A; CRP, C-reactive protein (normal range for SAA and CRP ≤ 10 mg/l)*.

### Molecular Investigations

Amplicon-based deep sequencing analysis revealed somatic *NLRP3* mutation in eight patients (Table 1). Four novel variants were identified: p.Y563C in three unrelated patients (patient 1, 2, and 3) with a mean mutant allele frequency (MAF) of 5.1, 11.1, and 3.2% respectively; p.G569V in patient 5 with a MAF of 21.1%; p.E567Q in patients 7 with a MAF of 15%, and p.G564D with a MAF of 5%. Two previously described amino acid substitutions were detected: p.A352T and p.E567K in patients 4 and 6, respectively, with a MAF of 14.6 and 5.4%.

**Figure 1:**
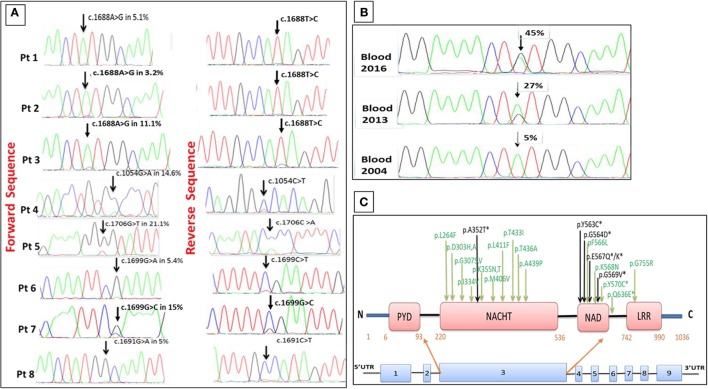
Somatic *NLRP3* mutations identified in eight late-onset cryopyrin-associated periodic syndrome (CAPS) patients. **(A)** Sanger chromatograms showing somatic *NLRP3* mutations and mutant allele frequency (MAF) in each case. **(B)** An increase in the MAF from 5.1 to 27 and 45% detected in blood samples collected 9 and 12 years apart in patient 1. **(C)** Schematic representation of the *NLRP3* gene and the encoded cryopyrin protein showing the location of all mosaic mutations identified in this study (in black) and from previous publications (in green), mutations identified in all late-onset-mosaic CAPS patients are shown by *.

In patients 3, 4, and 5, Sanger chromatograms revealed small peaks underneath the wild-type nucleotide in the relevant positions of the *NLRP3* gene suggesting mosaicism, while in the remaining patients with MAF ≤5% the small peaks were initially regarded as background signal (Figure 1A).

### Analysis of Tissue Distribution of the *NLRP3* Mutations

The frequency of the mutant allele was established by ADS in DNA extracted from whole blood, from isolated lymphocytes and myeloid cells, and from buccal epithelial cells, saliva, and urine (Table 2). The MAF was noticeably highest among neutrophils and monocytes (median 12.3 and 11%, respectively) when compared to other DNA sources (median 4.9%).

**Table 2:** Frequency of the *NLRP3* mutations identified among different cell populations established by amplicon-based deep sequencing.

Isolated cells	Patient 1 p.Y563C	Patient 2 p.Y563C	Patient 3 p.Y563C	Patient 4 p.A532T	Patient 5 p.G569V	Patient 6 p.E567K	Patient 7 p.E567Q	Patient 8 p.G564D	Control p.A439V
MAF (%) in whole blood	5.1^a^; 27^b^; 45^c^	3.3	11.1	14.6	21.2	5.4	15	5.0	56.3
MAF (%) in neutrophils	36.2^b^	7.4	10.7	16.2	21.8	12.3	ND	ND	53.2
MAF (%) in monocytes	44.8^b^	6.6	10.6	14.9	23.2	11.0	ND	ND	55.2
MAF (%) in B cells	15.4^b^	0.6^d^	6.6	12.3	17	7.1	ND	ND	54.2
MAF (%) in T cells	11.4^b^	0.4^d^	1.6	4.8	1.5	1.6	ND	ND	52.1
MAF (%) in saliva	20.5^b^	6.1	12.3	14.9	14.3	3.1	ND	ND	54.4
MAF (%) in buccal cells	20.5^b^	1.7	2.7	7.2	3.9	0.7^d^	ND	ND	47.1
MAF (%) in DNA isolated from other sources	0.6 in urine^d^	None	None	None	None	0.4 in urine^d^	None	None	None

*In Patient 1, DNA was isolated from blood samples obtained in ^a^2004; ^b^2013; and ^c^2016*.

*^d^Results under the threshold of 1% were considered to be false positives*.

*ND, not done*.

Our control for this study was an adult patient who was heterozygous for the *NLRP3* p.A439V mutation, which was inherited from her affected mother. The MAF was established at about 50% in all isolated cells (Table 2) and is consistent with the expected frequency of a germline heterozygous variant.

### Dynamics of the MAF in a Patient with Progressive Disease

Patient 1 had been healthy until age 46 years when she presented with symptoms consistent with CAPS, which became progressively more severe during 12 years of follow-up and required progressive up-titrations of her IL-1 inhibitor treatment. We performed a time-course study using blood samples obtained at the initial assessment in 2004 and subsequently in 2013 and 2016. ADS analysis revealed an increase in the MAF from a baseline of 5.1% (2004), rising to 27% (2013) and 45% (2016). These data were in concordance with the results obtained with the Sanger method of DNA sequencing (Figures 1B). In this patient, the c.1688A > G transition among purified B and T lymphocytes, neutrophils and monocytes had frequencies of 15, 11, 36, and 45%, respectively, on the samples collected in 2013. Thus far, we have not observed this phenomenon in other patients from this cohort, noting that they have been followed up for a median of only 3 years to date.

### Detection of ASC Protein Aggregates and IL-1β in the Serum

**Figure 2:**
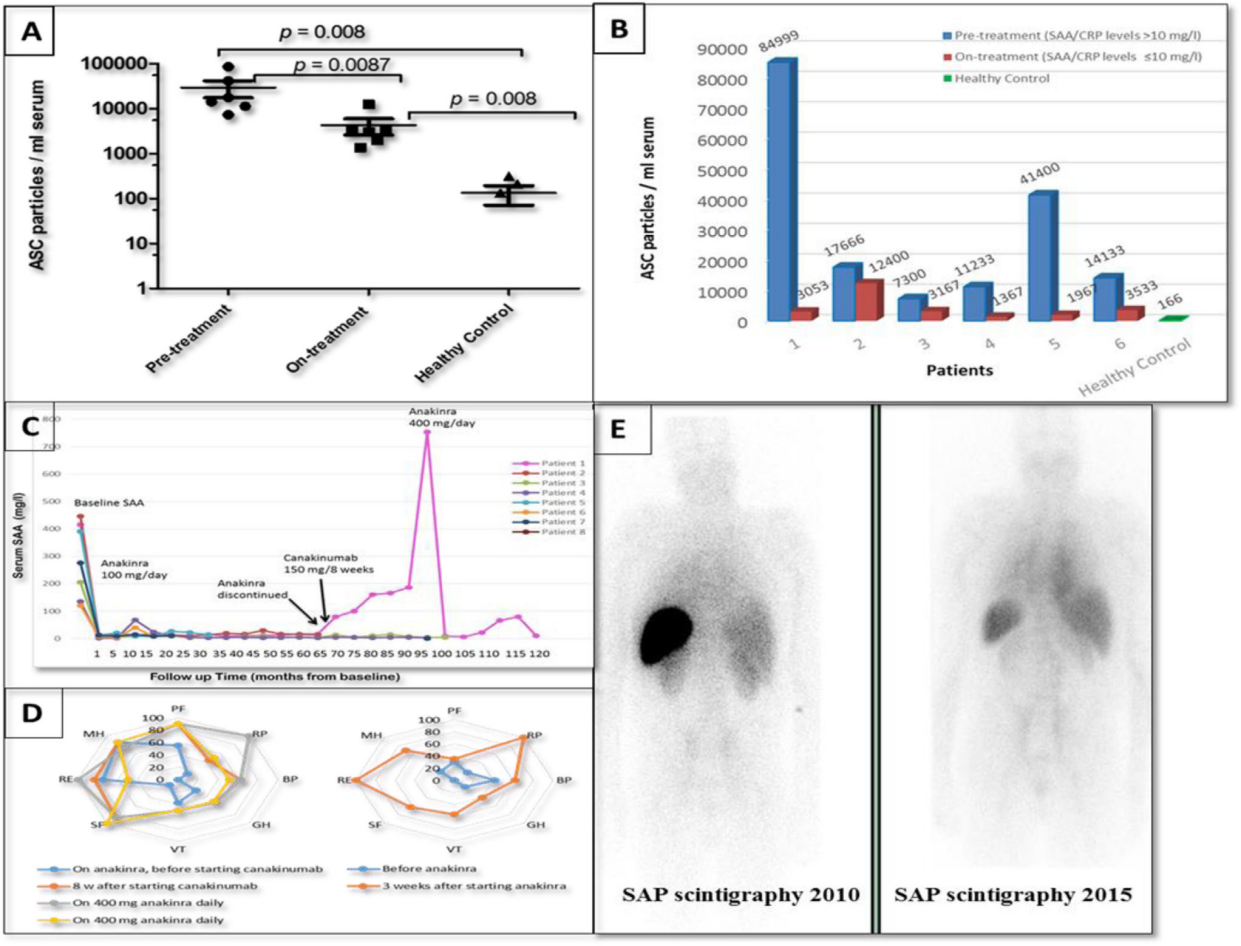
Response to IL-1 treatment in the eight patients with late-onset cryopyrin-associated periodic syndrome (CAPS) and somatic *NLRP3* mosaicism. **(A,B)** Extracellular ASC protein aggregates measured in the serum of six patients collected before introduction of IL-1 blocking therapy (anakinra 100 mg/daily) and while on treatment (only serum samples with the SAA/CRP levels ≤10 mg/l were selected) compared to ASC specks measured in the serum obtained from healthy volunteers. **(C)** Response to IL-1 treatment in the eight patients with late-onset CAPS showing a reduction in the serum amyloid A protein (SAA) levels; in patient 1 anakinra was discontinued and 150 mg/8 weekly of canakinumab was introduced resulting in a massive disease flare-up. **(D)** Quality of life before and while on treatment with anti-IL-1 treatment. Left panel: data from patient 1 who had relentlessly worsening clinical CAPS and required increasing doses of IL-1 inhibitor. Right panel: data from the remaining seven subjects; a comparison of the mean scores in each domain before and on-treatment shows improvement in seven domains. PF, physical function; RP, role physical; BP, bodily pain; GH, general health; VT, vitality; SF, social function; RE, role emotional; MH, mental health. **(E)** Posterior images of the whole body radiolabeled serum amyloid P component (SAP) scintigraphies performed in patient 3 showing regression of amyloid deposits; on the left is a SAP scan taken during the first assessment and on the right a SAP scan obtained at a follow-up visit 5 years later.

Extracellular ASC protein aggregates were measured in the serum collected from six patients with somatic *NLRP3* mutations before and during the administration of IL-1 blocking therapy (at different intervals from 1 to 12 months after starting treatment and up to 6 years in patient 1). A significantly higher abundance of ASC aggregates was detected in the serum of untreated patients when compared to the healthy volunteers (*p* = 0.008). Following effective IL-1 inhibition, serum ASC levels gradually decreased over time in patients whose inflammation remained suppressed (SAA/CRP levels ≤ 10 mg/l) (Figures 2A,B). In patient 1, who initially showed a good response to anakinra, the ASC concentration fell from pre-treatment values of 84,999 to 933/ml 6 months after treatment was commenced. The patient has subsequently relapsed, and the ASC specks levels measured over a 6-year period fluctuated between 1,500 and 5,333/ml. In all plasma samples analyzed, IL-1β concentration was below the detection threshold of the ELISA used in this study, corroborating previous reports (6, 20) where IL-1β in serum was found very low in serum samples of CAPS patients independently of disease activity.

### Laboratory and Clinical Response to IL-1 Blocking Therapy

All patients demonstrated remarkable clinical and biochemical responses to treatment with the recombinant IL-1 receptor antagonist (IL-1RA), anakinra (Kineret) administered by subcutaneous injections (100 mg/day) (Figure 2C). Overall, the quality of life had improved in our cohort (Figure 2D), including resolution of fever, headache, skin, and joint symptoms associated with a marked reduction of inflammatory markers, audiometric testing demonstrated hearing improvement in two patients (Patients 1 and 3). Renal function in patients 3 and 4 with AA amyloidosis gradually improved over time; proteinuria resolved in both cases in association with regression of amyloid measured by SAP scintigraphy (Figure 2E). Patient 5, notable for having developed CAPS in his eighth decade, sadly died of pancreatic cancer 18 months after diagnosis and highly effective treatment of CAPS.

Patient 1 had a complex clinical course. The patient remained in remission for almost 6 years after anakinra was initiated. However, due to breakthrough symptoms, her anakinra therapy was progressively intensified to 300 mg/day. An attempt to switch from anakinra to the anti-IL-1β monoclonal antibody canakinumab (150 mg/8 weeks) resulted in a major disease flare requiring hospital admission for sterile meningitis. Reintroduction of anakinra, up to 400 mg/day, in combination with prednisolone 5–10 mg/day initially brought her CAPS back under control for a period; a further relapse responded partially to the addition of canakinumab to this treatment regimen. Most recently, still further disease progression has been well suppressed though a combination of IL-1 and IL-6 blockade comprising anakinra 300 mg/day plus tocilizumab 4 mg/kg fortnightly. No serious infectious adverse events have occurred during 6 months of this novel combination anti-cytokine therapy.

## Discussion

Cryopyrin-associated periodic syndrome is a very rare disease with an estimated prevalence of 1–2 cases per million. The causative *NLRP3* gene mutation is usually inherited and, as a result, inflammatory symptoms typically become evident very early in life. CAPS is rarely considered among patients who present with compatible features in adult life (21). We describe here eight patients who developed CAPS at the median age of 50 years, with clinical features indistinguishable from inherited forms of the disease, including CNS inflammation and progressive sensorineural hearing loss. Schnitzler syndrome, which can mimic CAPS, was excluded through an array of sensitive paraprotein studies. ADS analysis revealed somatic *NLRP3* mutations in all cases. Two mutations (p.A352T and p.E567K) have previously been reported in two Japanese children diagnosed with MWS associated with somatic *NLRP3* mosaicism (12) and in a single heterozygous patient with CINCA-NOMID assessed in our center. Four novel mutations were discovered: p.Y563C, interestingly present in three unrelated subjects, p.G564D, p.E567Q, and p.G569V. These new mutations have not been reported in the 1000 Genomes Project database and in the Exome Aggregation Consortium, suggesting their absence among the healthy population. The discovery of p.Y563C in three unrelated patients is particularly intriguing; the tyrosine amino acid in residue 563 is relatively well conserved among different species, and a different missense mutation at this residue (p.Y563N) has been reported as a cause of CAPS in two patients (21). The *NLRP3* mutations identified in our cohort and in the two previously reported late-onset mosaic CAPS cases (13, 14) are located in close proximity within the NAD domain, implying this part of the gene might be a hotspot for somatic mutations (Figure 1C). This suggests that it is important to conduct a meticulous analysis of this part of the *NLRP3* gene in all subjects who present in adult life with symptoms consistent with CAPS.

Somatic mosaicism is well described in the molecular pathology of cancer and neurodegenerative diseases and recently has gained recognition as a cause of AIDs, including CAPS (8–14), SAVI syndrome (22), Blau syndrome (23, 24), and TRAPS (25). Thus far, studies of all mosaic CAPS patients (8–14) have shown that the timing of when the mutation occurs and their subsequent selection can vary greatly. A mutation acquired during the early stages of embryogenesis will cause mosaicism affecting all cells of the body and, with respect to pathogenic mutations in the *NLRP3* gene, would be expected to be associated with onset of inflammatory disease in early life (8). By contrast, lineage-specific genetic variants indicate mutational events that have occurred at a later stage of fetal development or any time of life subsequently, resulting in tissue-limited mosaicism (26, 27). To investigate the timing, etiology and the dynamics of the mutational events in our patients, we examined DNA from a variety of different tissues, along with samples obtained serially over a median duration of 3 years (range 2–7) in five subjects and over 14 years in one patient. In one case, patient 2, the mutant allele was detected predominantly in cells of myeloid linage, which is consistent with the two previously reported late-onset CAPS cases caused by myeloid-restricted somatic *NLRP3* mosaicism (13, 14). Analysis of mesodermal (leukocytes), ectodermal (buccal epithelial) cells, and endodermal (urinary epithelial) cells in five patients identified the mutant allele in several cell types, but at differing frequencies. With regard to leukocytes, the MAF was higher among myeloid cells in comparison to lymphocytes, in particular T cells. A plausible explanation for the difference in the MAF among myeloid and lymphoid cells, despite a common progenitor, could be at least in part due to impairment of the lymphoid lineage differentiation and/or a selective increase of the myeloid lineage. Such lymphoid to myeloid shift is well documented in the bone marrow studies of elderly patients (28, 29). The low mutant allelic frequency seen in buccal cells is likely to represent contamination of the sample with leukocytes, also reported in other studies (14). Thus, we conclude that our results point to a truly late-onset mutational event at the level of a multipotent hematopoietic stem cell or common myeloid progenitor in the case of patient 2. Including the two previous reports (13, 14), there are now 10 patients diagnosed with CAPS in their mid-late adult life, caused by somatic NLRP3 mutations, but the precise mechanisms of this phenomenon are not known. Several studies have demonstrated that the incidence of somatic alternations increases with age (30) and can be caused by less effective DNA repair pathways and decrease in cellular diversity. On average, 1.3 ± 0.2 somatic exonic mutations are acquired per hematopoietic stem cell per decade (31). Hematopoietic progenitor and stem cells, such as stem cells in other tissues, accumulate somatic mutations throughout the human lifespan, most of these are nonpathogenic and without functional consequence or potential to contribute to clonal expansion. Certain mutations, however, confer a survival advantage to the mutated cell and allow clonal expansion. In our cohort, proliferation of the somatically altered cell population had reached detectable fraction and although we are unclear on the precise mechanisms, it may be possible that other, co-existing somatic alternations may have driven the expansion of mosaic clone.

We had the opportunity to re-analyze the *NLRP3* gene in patient 1, who is notable for having been the first “mutation-negative” CAPS patient that we had encountered, and to date the only patient whose inflammatory disease severity has inexorably worsened. Studies on blood samples obtained at presentation and nine and 12 years subsequently demonstrated an increase in the frequency of the mutant allele from 5.1 to 45% over this period, completely in keeping with her progressive requirement for intensification of IL-1 inhibiting therapy. Thus far, we have not observed this phenomenon in other patients in this cohort, all of whom have had shorter follow-up and none of whom have required up-titration of their highly effective therapy. The clonal expansion of myeloid cells evident in patient 1 remains unclear but a potential explanation is that the affected cells have a selective survival advantage. Clonal non-malignant expansion of hematopoietic stem cells has recently been described in large scale sequencing studies in healthy adult subjects, in whom the frequency of clonal hematopoiesis increased from 1% in those over age 40 to about 10% by age 65 years (30, 32). The mechanism might involve somatic mutations in several cancer related genes which confer advantages to hematogical stem/progenitor cells such as cell renewal and clonal expansion (33), but these “driver mutations” do not seem to lead directly to hematopoietic malignancies (30). Given the high frequency of this phenomenon in the older population, we hypothesize that one or more of these “driver mutations” could have led to the apparent selective survival advantage by clonal expansion of *NLRP3* mutated cells of the myelo-monocytic line in our patient. Stepwise progression occurs in other non-malignant clonal disorders, commonly including monoclonal gammopathy of undetermined significance, and we await with great interest longer term follow-up in our other patients.

Most of our patients had been very symptomatic for many years prior to diagnosis of CAPS, which facilitated their treatment with IL-1 blockade. As is the case in CAPS generally, inhibition of IL-1 was magnificently effective in this cohort with late-onset disease, by and large completely suppressing their inflammatory disease. End organ damage improved in four cases, comprising hearing improvement in two patients and regression of AA amyloid-associated renal dysfunction in two patients. AA amyloidosis is a life-threatening complication of CAPS, which hitherto has only been reported in patients with germline *NLRP3* mutations. Amyloidosis complicating acquired CAPS is not inherently surprising since AA amyloid develops in a small proportion of all patients with a sustained acute phase response due to a longstanding inflammatory disorder, generally after 10–20 years duration, and low-level somatic *NLRP3* mosaicism is evidently a cause of this. The incidence of AA amyloidosis is notably high in patients with inherited AIDs because the inflammatory disorder is lifelong. Since the nature of the causative underlying inflammatory disorder is unclear in about one-fifth of patients who present with AA amyloidosis, and because CAPS can occur in the absence of characteristic rash, we recommend in-depth sequencing of all of the known inherited AID genes in all patients with AA amyloidosis of undetermined cause.

Despite CAPS being associated with a high level production of IL-1β, there is no correlation between active disease and circulating IL-1β, which is virtually undetectable in human plasma, even in patients with active CAPS (20). In our cohort presented here, we obtained an indirect evidence that CAPS is associated with an upregulated NLRP3-inflammasome activation by measuring levels of ASC aggregates in the patients’ serum. We have previously reported high circulating levels of ASC aggregates during inflammatory flares in CAPS patients with somatic *NLRP3* mutations (6) and describe here studies in our cohort before and during therapy with IL-1 inhibition. Extracellular ASC aggregates are important mediators of inflammation by recruiting and activating the NLRP3-inflammasome, including this pathway in *NLRP3* wild-type cells leading to significant amplification of IL-1β release (6, 34). In our patients, the serum concentration of ASC aggregates measured during active disease (before administration of IL-1 blocking therapy) was significantly higher compared to that of healthy volunteers. Following treatment with IL-1 blockade, the ASC protein concentration was initially stable but gradually decreased over a 12-month period in patients whose CAPS related inflammation remained well suppressed, i.e., in those whose SAA and CRP concentration remained in the reference range. In patient 1, who had a complex clinical course, the ASC concentration fell during the first 6 months of IL-1 blocking treatment, but subsequently became elevated again in association with her various clinical relapses. These data indicate that the abundance of circulating ASC aggregates reflects inflammatory disease activity in CAPS but that remission must be sustained for some months before a fall in concentration occurs.

In conclusion, this study has identified somatic *NLRP3* mosaicism as the etiology of late onset but otherwise typical CAPS in 8% of patients seen in the adult UK national CAPS treatment service. The mechanisms underlying the observed differing frequency of the mutant allele among lymphocytes and myeloid cells remains unclear, but may include selective survival advantage of certain mutated cell types and/or clonal expansion of myeloid cell lines that may be unrelated to the acquired *NLRP3* mutation. This study extends growing evidence that somatic *NLRP3* mosaicism is not an uncommon cause of CAPS, which should be considered in patients with CAPS-compatible symptoms that start late in life.

## Ethics Statement

This study was carried out in accordance with the recommendations of Royal Free Hospital and University College Medical School Research Ethics Committee (REC reference number 06/Q0501/42) with written informed consent from all subjects. All subjects gave written informed consent in accordance with the Declaration of Helsinki. The protocol was approved by the University College Medical School Research Ethics Committee.

## Author Contributions

DR and SG wrote the manuscript. DR, SG, ABaginska, HT, JA, EO, ABaroja-Mazo, and PP performed experiments, and analyzed and interpreted the data. HL, PB, and PH composed the final versions of the manuscript. TL, RW, HL, SS, and PH looked after patients described in this study. All the authors read and approved the final version of the manuscript.

## Conflict of Interest Statement

The authors declare that the research was conducted in the absence of any commercial or financial relationships that could be construed as a potential conflict of interest.
